# 5-ALA Fluorescence in Native Pituitary Adenoma Cell Lines: Resection Control and Basis for Photodynamic Therapy (PDT)?

**DOI:** 10.1371/journal.pone.0161364

**Published:** 2016-09-01

**Authors:** Andrei Nemes, Thomas Fortmann, Stephan Poeschke, Burkhard Greve, Daniel Prevedello, Antonio Santacroce, Walter Stummer, Volker Senner, Christian Ewelt

**Affiliations:** 1 Institute of Neuropathology, University Hospital Muenster, Muenster, Germany; 2 Department of Neurosurgery, University Hospital Muenster, Muenster, Germany; 3 Department of Radiotherapy-Radiooncology, University Hospital Muenster, Muenster, Germany; 4 Department of Neurological Surgery, Ohio State University, Columbus, United States of America; 5 Department of Neurosurgery, University Hospital Tuebingen, Tuebingen, Germany; Heinrich-Heine-Universitat Dusseldorf, GERMANY

## Abstract

Objective: Pituitary adenomas (PA), especially invasive ones, are often not completely resectable. Usage of 5-aminolevulinic acid (5-ALA) for fluorescence guided surgery could improve the rate of total resection and, additionally, open the doors for photodynamic therapy (PDT) in case of unresectable or partially resected PAs. The aim of this study was to investigate the uptake of 5-ALA and the effect of 5-ALA based PDT in cell lines. Methods: GH3 and AtT-20 cell lines were incubated with different concentrations of 5-ALA, protoporphyrin IX (PPIX) fluorescence was measured by flow cytometry and fluorescencespectrometry. WST-1 assays were performed to determine the surviving fraction of cells after PDT. PPIX fluorescence intensities and PDT effect of the pituitary adenoma cells were compared to U373MG, a well-known glioblastoma cell line. Results: Both cell lines showed a 5-ALA dependent intracellular PPIX fluorescence. Significant differences after 24hrs of incubation were observed in AtT-20 cells in comparison to GH3. Regardless of the incubation or metabolism time, there was a proliferation inhibiting effect after PDT, with no statistical significance. Conclusion: Since GH3 cells showed a heterogenous uptake of 5-ALA in the flow cytometry profile, but not constantly high concentrations they might have a 5-ALA efflux mechanism, which still needs to be determined. In the case of AtT-20, the cells might need a longer time for the uptake due to their size or slow metabolism. We showed that the different cell lines have different uptake and metabolism mechanisms, which needs to be further investigated. The general uptake of 5-ALA allows the possibility of resection control and PDT for pituitary adenomas. But, the role of PDT for unresectable pituitary adenomas deserves further investigations.

## Introduction

Pituitary adenomas have an incidence of 10–12% of all intracranial tumors and are mostly benign tumors of the anterior pituitary gland [1]. While the majority of pituitary tumors include slow growing, non-metastasizing adenomas, approximately one third will become invasive and a very small proportion (0.1%) will become malignant [2]. Many factors influence the proliferation of pituitary adenomas, such as angiogenesis, apoptosis, growth factors, oncogenes, tumor suppressor genes, and hormone receptors [2,3]. Treatment of choice for pituitary adenomas has traditionally been the transnasal, transsphenoidal surgical resection, either via microscope or endoscope, whereas in prolactinomas and selected patients with acromegaly, medical treatment is a well-established alternative [4,5]. Gross total resection especially in invasive adenomas is not always possible. Adjuvant treatment modalities, such as dose fractionated radiotherapy, specific drug therapy or, particularly in the last ten years, radiosurgery are required [6].

5-aminolevulinic acid (5-ALA) is a natural biochemical precusor of hemoglobin, synthesized from the amino acid glycin and succinyl-CoA, that induces synthesis and accumulation of fluorescent porphyrins, mainly protoporphyrin IX, in various epithelia and cancerous tissues [7]. Administered 3 hours prior to anaesthesia it leads to an accumulation of PPIX in malignant gliomas that functions as a photosensitizer [8,9]. Intraoperative excitation of PPIX and visualization of the resulting fluorescence isimplemented through a modified neurosurgical operating microscope. Consisting of a connectible light source (375-440nm) and a 440nm long pass filter for the emitted light [9,10] it enables a good discrimination between neoplasm and healthy brain tissue.

Photodynamic therapy (PDT), which is an emerging alternative treatment strategy for various types of cancer [11–13], utilizes another physical property of PPIX. The combination of oxygen, red light (635nm) and 5-ALA based PPIX as photosensitizer leads to the generation of highly reactive oxygen species, particularly singlet oxygen, followed by selective cell death [14,15,16]. First aim in our in-vitro experiments with pituitary adenoma cells was to prove the uptake of 5-ALA into tumor cells in order to provide a resection control during surgery. Second aim was to show the effect of 5-ALA and PDT on cell viability for providing another, more selective irradiation modality for not completely resectable adenomas. To our knowledge, this issue has not been shown in literature so far.

## Material and Methods

### Reagents

5-aminolevulinic acid hydrochloride was purchased from Fagron (Barsbüttel, Germany), methanol and acetone from Carl Roth (Karlsruhe, Germany), protoporhyrin IX from Sigma-Aldrich (Munich, Germany) and tetrabutylammonium dihydrochloridephosphate from AppliChem (Darmstadt, Germany). Cell culture medium DMEM/F-12, fetal bovine serum (FBS), Trypsin-EDTA and PBS were purchased from Gibco/LifeTechnologies (Darmstadt, Germany). Penicillin and streptomycin were purchased from Biochrom (Berlin, Germany) and the Wst-1 Assay was purchased from Roche (Mannheim, Germany).

### Cell Culture

The pituitary adenoma cell lines GH3 and AtT-20 (both from ATCC, Manassas, Virginia, US) and the glioblastoma cell line U373 MG (from the original cultures established in Uppsala 1968 [17]) were cultured as adherent monolayer at 37àC in a 5% CO2 humidified atmosphere. Cells were grown in DMEM/F-12 supplemented with 17% FBS, 100 U/ml penicillin and 100 g/ml streptomycin and DMEM supplemented with 10% FBS, 100 U/ml penicillin and 100 g/ml streptomycin, respectively. Medium was changed every third day and cells were harvested when they reached 80–90% confluence with 0.25% Trypsin-EDTA for further experiments.

### Flow Cytometry

For PPIX fluorescence analysis 1x10^5^ cells were seeded into 6 well plates and incubated overnight till completely adherent. The next day cells were incubated with 100 g/ml 5-ALA or medium alone for 6 or 24hrs. Subsequently, the cells were washed twice with PBS, harvested and centrifuged at 350rcf for 5min. The supernatant was aspirated and the cells were resuspended in 1ml PBS. Fluorescence measurements were performed with a CyFlow Space flow cytometer from Partec (Muenster, Germany). Living cells were determined and gated according to the forward scatter/ side scatter (FSC/SSC) profile. The cells were excited at a wavelength of 375nm and the emission was measured with a 630nm long pass filter. Mean fluorescence intensities MFI were calculated by subtracting the control geometric mean fluorescence from the 5-ALA condition.

### Protoporphyrin IX Quantification

Quantifications of intracellular PPIX levels were determined via spectrofluorometrical measurement. Therefore, 1x10^6^ cells were seeded in a T25 cell culture flask. Approximately 12hrs later cells were treated for 6 or 24hrs with 100 g/ml 5-ALA or media alone. After the incubation period the cells were washed, trypsinized and centrifuged at 350rcf for 5min. The supernatant was removed, cells were resuspended in 1ml PBS, transferred in 1.5ml tubes and centrifuged again. PBS was removed and cell pellets were frozen at -80°C till further usage. The frozen cell pellets were thawed on ice in 1ml of 50mM tetrabutylammonium dihydrogenphosphate in acetone/methanol (1:1 v/v). Samples were treated with ultrasonic waves for 1min. In avoidance of heating up the samples the sonification was split up in 15s intervals. Subsequently, the samples were stored for 10min in the dark and centrifuged at 11000 rcf for 5min. The supernatant was used for the spectrofluorometrical measurement [18] in a FluoroMax II (Horiba Scientific, Unterhachingen, Germany) and excited at 405nm. The spectra from 450nm to 750nm was analyzed. For each experiment the untreated condition was subtracted from the 5-ALA treated measurement.

### Cell Size Determination

Phase contrast images of adherent cells were taken with an Olympus IX50 microscope and an Olympus ColorView III camera (Olympus, Hamburg, Germany). The cell surface was calculated with the region of interest (ROI) manager tool of Fiji (Fiji is just ImageJ, http://fiji.sc/Fiji, [19]). For all cell lines the size of 10 cells from different passages was calculated and averaged.

### Photodynamic Therapy

Ten thousand (1x10^4^) cells were seeded in a black-wanded 96 well plate (Greiner Bio One, Frickenhausen, Germany) in quadruplicates. The following day cells were treated with different concentrations of 5-ALA (25, 50, 75, 100, 125, 150, 175, 200 μg/ml) or media alone for 6 and 24hrs. Beside, control cell line U373 was incubated for PDT in 25 μg/ml 5-ALA. Subsequently, the media was removed, washed once with PBS and replaced by 100 l standard culturing media. Irradiation was performed with a Ceralas 635-nm PDT diode laser (Biolitec, Jena, Germany) at 635nm with 25 J/cm^2^ for 250s and with a defined distance of 9.3 cm between the diffusion lens and the 96-well plate.

### Viability Assay

Twelve to fourteen hours after light irradiation the Wst-1 assay was performed according to the manufacturing protocol. Briefly, 10 l Wst-1 reagent was added to 100 l media per well of the 96 well plate and incubated for 2hrs in the cell culture incubator. Plates were shaken for 30s and absorbance was measured at 440nm, background measurement was performed at 650nm.

### Statistics

All experiments were performed at least three times. Statistics were performed with Graph Pad Prism. The significance was determined using one way ANOVA comparing the groups irradiated/non-irradiated or 6hrs/24hrs of each experiment followed by bonferroni’s multiple comparison test. P≤0.05 was considered significant. Error bars represents the standard error of the mean (S.E.M.).

## Results

### Cellular PPIX Fluorescence

**Fig 1:**
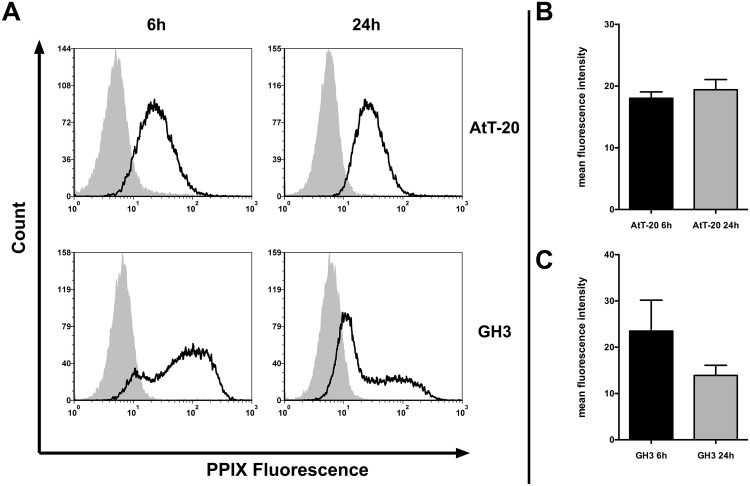
Flow cytometry data of PPIX fluorescence in pituitary adenoma cells. (A) The pituitary adenoma cell lines GH3 and AtT-20 were incubated for 6 and 24hrs with 100 g/ml 5-ALA (black line) or with media alone (gray area). One out of 3–7 independent experiments is shown. (B andC) MFI of all flow cytometry experiments are shown. Error bars represent S.E.M.

Essential parameters for PDT are the amount of intracellular PPIX, availability of oxygen and red light. Under monolayer culture conditions and performing the experiments at atmospheric pressure the limiting factor is the intracellular PPIX accumulation. We analyzed this by flow cytometric and spectrofluorimetrical measurements in two pituitary adenoma cell lines: GH3 and AtT-20 and the glioblastoma cell line U373MG. Flow cytometry data of both pituitary adenomas has shown a shift in the positive area compared to untreated cells (Fig 1A), with mean percent positive events after 6hrs of 51.4% and 62.6% in GH3 and AtT-20, respectively (Table 1). Comparing the MFI in our cell lines showed no statistical significance regarding the comparison of 6 vs. 24hrs or AtT-20 vs. GH3 (Table 1; Fig 1B and 1C). The glioblastoma cell line U373 MG is known for strong 5-ALA uptake and metabolism to PPIX and showed a good photodynamic reaction upon 5-ALA based PDT [16,18]. Therefore, we decided to measure the intracellular PPIX fluorescence in these cells as a positive control. Flow cytometric analysis revealed 97.4% positive events and an MFI of 46.05 of all gated cells (Table 1) in U373MG after 6hrs of incubation.

**Table 1:** Comparison of flow cytometry, spectrofluorometer data and cell size. Comparison of flow cytometry, spectrofluorometer data and cell size in GH3, AtT-20 and U373MG cell lines after 6 and 24hrs of 5-ALA incubation. FACS as percent positive events n = 3–7. MFI as mean fluorescence intensity n = 3–7. Spec. as arbitrary units n = 3–4. Cell size n = 10

	GH3		AtT-20		U373MGS
	6hrs	24hrs	6hrs	24hrs	6hrs
**FACS [% +/- S.E.M.]**	51.4 (10.3)	38.4 (5.1)	62.6 (4.9)	76.2 (4.4)	97.4 (2.6)
**MFI [+/- S.E.M.]**	23.5 (6.68)	13.9 (2.19)	18.3 (1.03)	19.4 (1.66)	46.05(6.1)
**Spec [a.u.]**	147046	152168	112295	250347	200566
**Cell size [μm^2]**	746.5		1094.5		2575.9

**Fig 2:**
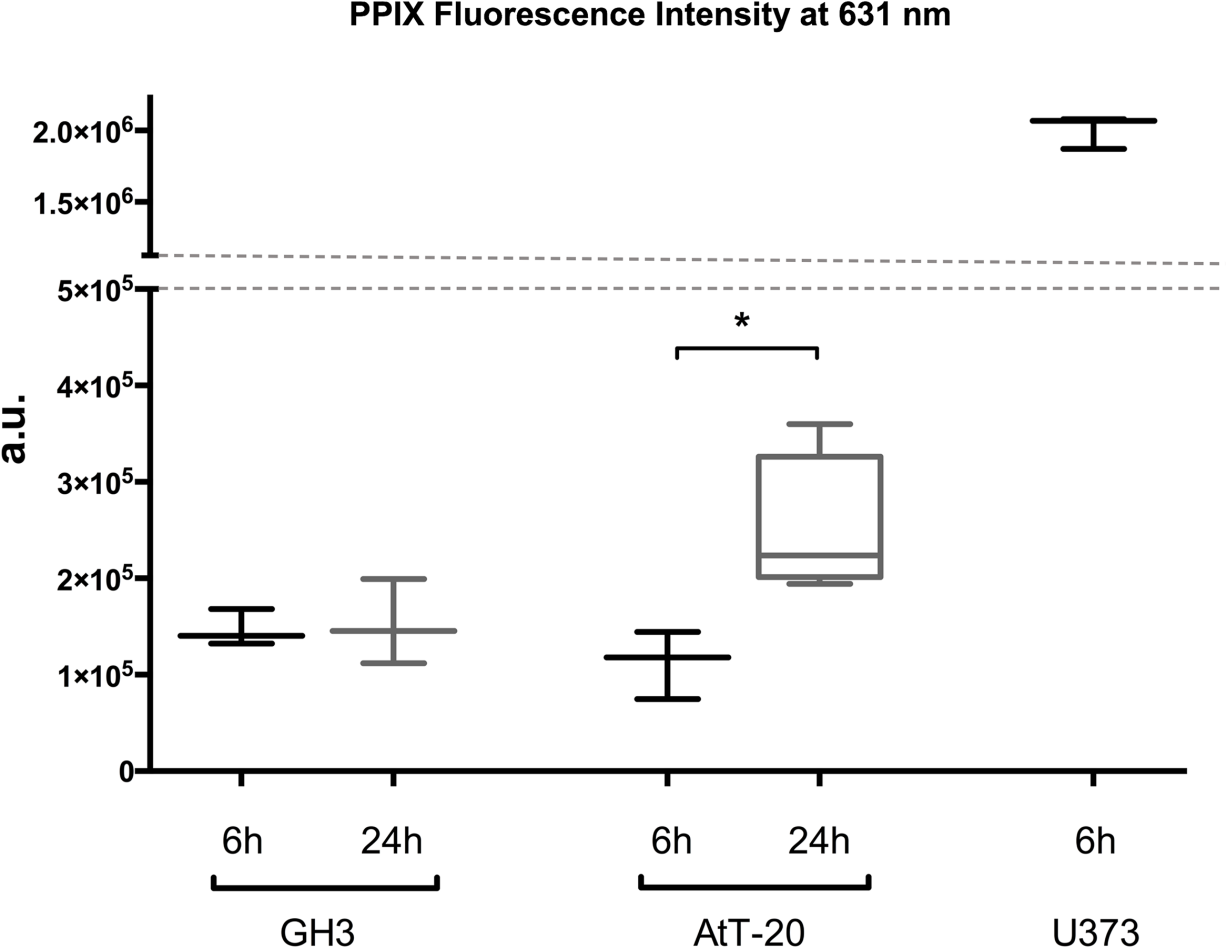
Spectrofluorometric measurment of GH3, AtT-20 and U373 MG cell lysates after excitation at 405nm. 1x10^6^ cells were seeded and incubated with or without 5-ALA for 6 and 24hrs. The spectra from 450nm to 750nm was analyzed. For each experiment the untreated condition was subtracted from the 5-ALA treated measurement. Peak values at 631nm are compared after 6 and 24hrs of incubation in all three cell lines as arbitrary units (a.u.). Three to four independent experiments were performed and presented as box plot with min.-max. whiskers, one way ANOVA followed by Bonferroni’s post-hoc analysis was used for evaluating the statistical significance, * indicates p ≤ 0.05.

Because of the fact that our pA cell lines showed a slow growth rate compared to glioblastoma cell lines, we next asked what will happen if you prolong the 5-ALA incubation time in these cells. After an incubation period of 24hrs we could not see any statistical differences measured by flow cytometry (Table 1 and Fig 1B and 1C). GH3 cells showed a slightly weaker signal after 24hrs of incubation compared to the 6hrs treatment (Fig 1C). In contrast to the FACS results, the spectrofluorometrical analysis of AtT-20 showed a significant increase in the PPIX fluorescence after 24hrs (Fig 2). Protoporphyrin IX fluorescence signal in U373MG was by far more intense compared to the pituitary adenoma cells. Spectrofluorometrical measurement showed a 13.6 and 17.7 times higher signal in U373MG compared to GH3 and AtT-20, respectively. Because of the fact that the spectrofluorometrical measurement represents the pooled intensity of 1x10^6^ cells we determined the average size of the cell lines at adherent conditions. We analyzed it by phase contrast images followed by calculation of the surface with the ROI Manager tool in Fiji (Table 1). AtT-20 is 1.5 times larger than GH3. U373MG cells are 2.4 times the size of AtT-20. Regarding the fact that U373MG cells are slightly larger than AtT-20 but showed a 13.6 times higher PPIX fluorescence indicates that our pA cell lines were not saturated in their intracellular PPIX concentrations.

### Photodynamic Therapy

**Fig 3:**
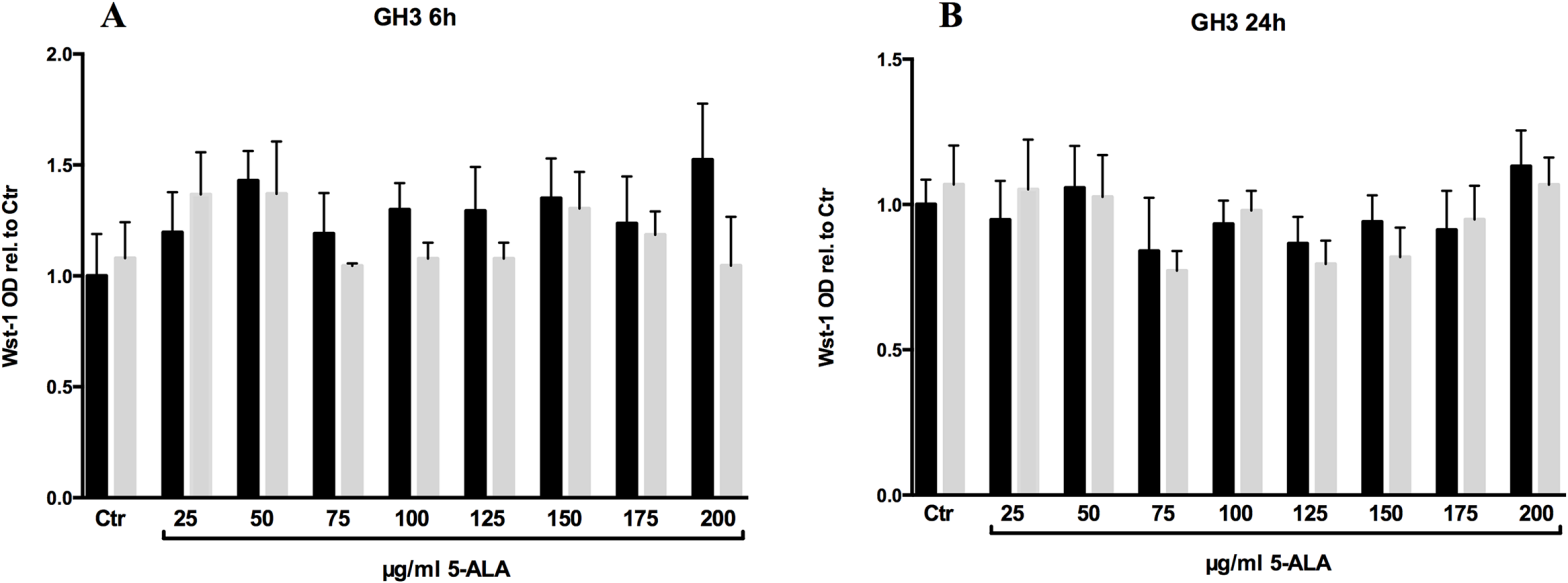
Relative cell viability of GH3 cells. Cells were monitored with Wst-1 assay and compared to the untreated control. The cells were pretreated with different concentrations of 5-ALA for 6 (A) and 24hrs (B). Subsequently, the cells were irradiated with red light (635nm) for 250s and 25 J/cm^2^ (gray bars) or left non irradiated (black bars). Four independent experiments were performed and averaged. Error bars indicates S.E.M., no statistical significance was found as calculated by one way ANOVA followed by Bonferroni’s multiple comparison test.

**Fig 4:**
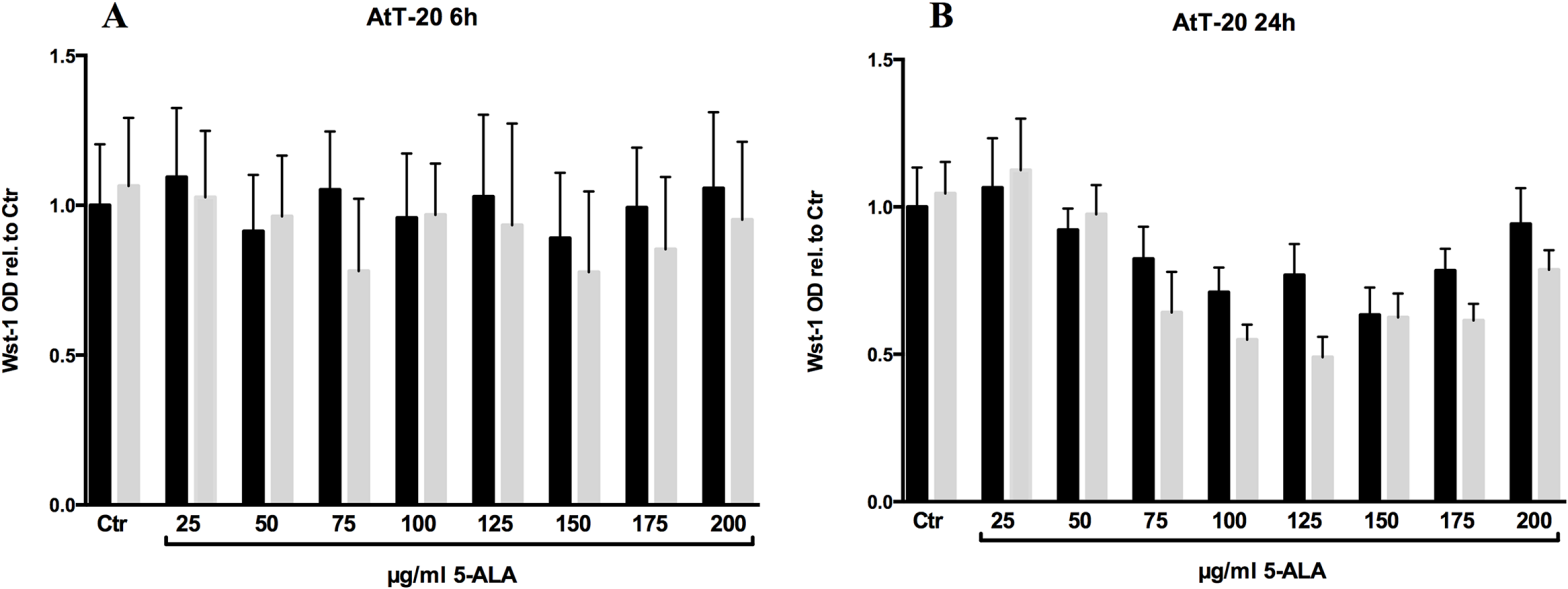
Relative cell viability of AtT-20 cells. Cells were monitored with Wst-1 assay and compared to the untreated control. The cells were pretreated with different concentrations of 5-ALA for 6 (A) and 24hrs (B). Subsequently, the cells were irradiated with red light (635nm) for 250s and 25 J/cm^2^ (gray bars) or left non irradiated (black bars). Four independent experiments were performed and averaged. Error bars indicates S.E.M., no statistical significance was found as calculated by one way ANOVA followed by Bonferroni’s multiple comparison test.

**Fig 5:**
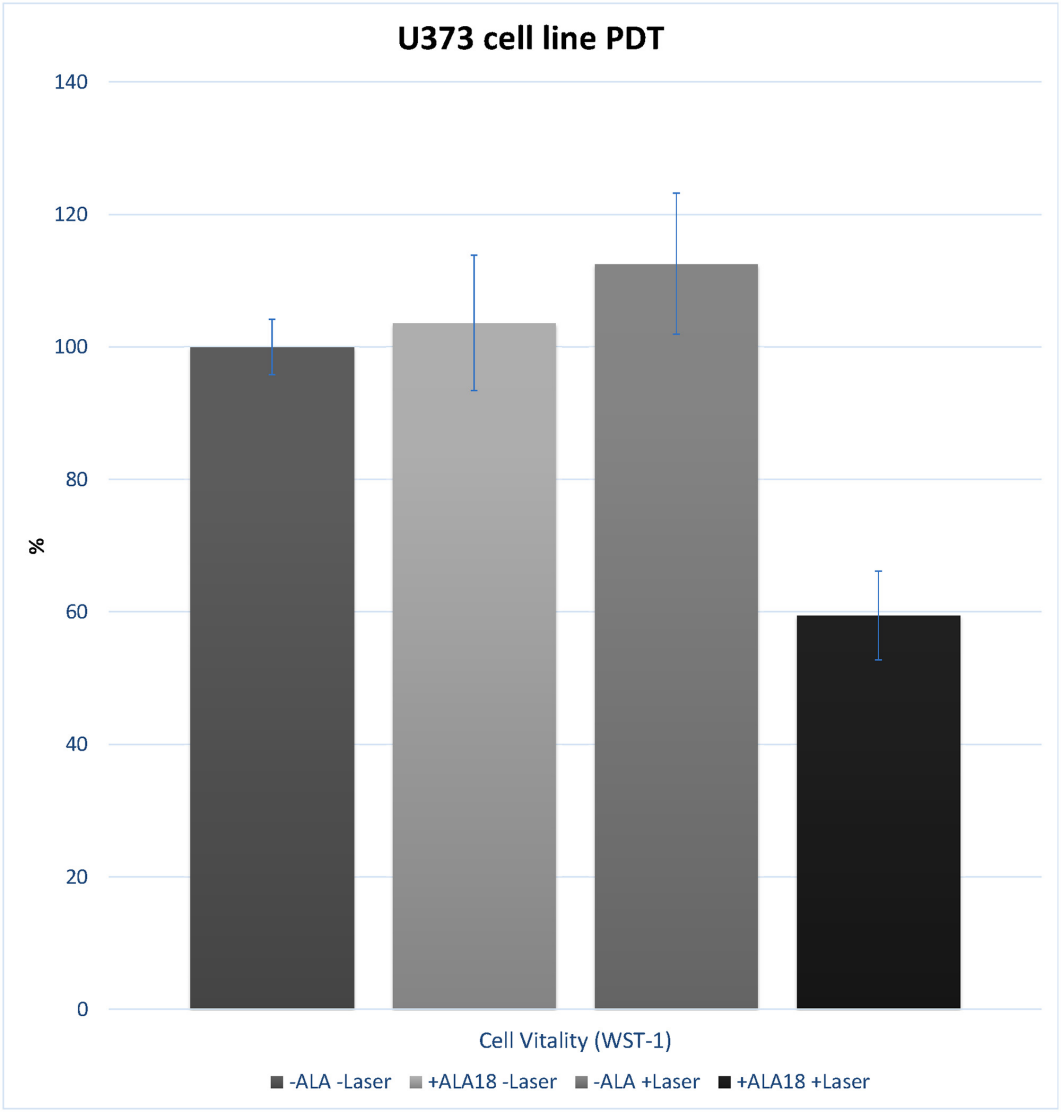
Relative cell viability of U373 cells. Cells were monitored with Wst-1 assay and compared to the untreated control group without 5-ALA and laser, with 5-ALA and without laser, without 5-ALA and with laser. The cells were treated with 25 μg/ml 5-ALA for 6 hours (n = 12). Subsequently, the cells were irradiated with red light (635nm) for 250s and 25 J/cm^2^. Error bars indicates S.E.M., statistical significance of p<0.05 was found for treated cell group with 5-ALA and with laser.

After the intracellular PPIX accumulation was verified in the two cell lines included in this study, we tested whether the amount of the photosensitizer is sufficient to induce a photodynamic reaction. Therefore, we incubated AtT-20 and GH3 cell lines with different concentrations of 5-ALA (25 μg/ml—200 μg/ml) for 6 and 24hrs and irradiated the cells with red light (635nm) for 250s and 25 J/cm2. Twelve to fourteen hours later, we performed a metabolism based viability assay (Wst-1). Regardless of whether you increase the 5-ALA concentration or the incubation time, combined with light irradiation the GH3 cells were not significantly affected by this treatment (Fig 3). Once more we performed the same experimental setup and analyzed the reaction of AtT-20 cells upon 5-ALA based PDT. After 6hrs of 5-ALA treatment, no significant change in the survival of the cells was detectable (Fig 4). This extends to the 24hrs data, with the exception that a general decline in Wst-1 assay signal, starting at a 5-ALA concentration of 75 μg/ml, is measurable in both conditions, irradiated and non-irradiated. U373 cells showed a significant reaction on PDT after incubation in 25 μg/ml 5-ALA for 6 hours (Fig 5). The concentration of 25 μg/ml 5-ALA is experienced to be effective in former experiments as sufficient control dose for U373 cell line.

## Discussion

In this study we demonstrated the uptake and metabolism of 5-ALA in two pituitary adenoma cell lines, GH3 and AtT-20. Photodynamic therapy combined with different 5-ALA concentrations (up to 200 μg/ml) and two incubation periods (6 or 24hrs) showed no significant effect on the survival of these cells in vitro.

Surgical tumor resection is considered to be the first treatment modality of drug-resistant pituitary adenomas that are completely or partially resectable [20–23]. Thanks to recent developments in endoscopic instruments and techniques, several authors proposed a fully endoscopic approach to the sellar region [24,25] for a better visualizing, but the discussion about effect and transmission rate remains controversial [26,27]. Failure of surgery of invasive pituitary adenomas could be minimized when a visualization of adenoma tissue invading the cavernous sinus or the suprasellar region was provided. Our proposal in this experimental setting was to establish a new diagnostic tool in pituitary adenoma surgery by using 5-ALA in order to distinguish between normal tissue and tumor tissue and therefore, to improve cure rate and preservation of pituitary function. So far, there is only one observational study in the literature in which the authors used 5-ALA applied pre-operatively for visualizing tumor tissue (optical biopsy system) by putting a laser diode into the sellar cavity during surgery and by measuring the light emission by photodiagnostic filters in 30 patients [28]. They could find that this is a feasible and reliable way to localize the 175 adenomas and may lead to improved outcome. But what really emitted intra- or extra-cellularly the fluorescent light was not clear. Basic research for 5-ALA derived fluorescence-guided surgery in pituitary adenomas is required.

The non-uniform PPIX accumulation in our two cell lines leads to several possible interpretations. On one side (i) the metabolism rate of pituitary adenoma cell lines is slow and due to the larger cell size AtT-20 took longer to become saturated. (ii) The majority of GH3 cells have an additional PPIX efflux mechanism that constantly discharges PPIX out of the cell that leads to a fixed PPIX signal indifferent of a longer incubation time. (iii) GH3 and AtT-20 represent two distinct pituitary adenoma entities, prolactin and somatotrophin and adrenocorticotropic hormone (ACTH) producing adenoma, respectively. Taken together these findings show that pituitary adenoma cell lines have a varying PPIX fluorescence pattern among themselves and in-between different cell lines.

The 5-ALA-based PDT causes mitochondrial and nuclear DNA damage [29] and apoptosis occurs due to mitochondrial release of cytochrome c, endoplasmatic reticulum stress, decreases in Bcl-3 and Bcl-xL, and activation of caspase-9 and caspase-3 [15,30]. In our experiments, we could confirm a significant effect of PDT in combination with 5-ALA in U373 glioma cell suspension (Fig 5). Moreover, a recent in-vitro study by Etminan and colleagues concluded that 5-ALA/PDT has a long-lasting effect on glioblastoma cells, affecting their migratory and invasive activity, possibly mediated by changes induced in the cytoskeleton and the expression of molecules involved in matrix invasion [16]. The 5-ALA-derived fluorescent treatment option with excitation at 635nm by a laser diode does not only eliminate glioblastoma cells directly, it might as well, target the tumor vasculature, and result in an anti-tumoral immune response [31,32]. Additionally, it affects one of the most significant characteristics of glioblastomas, their invasiveness. This hypothesis was confirmed by other authors. Li and colleagues demonstrated regional and systemic anti-tumor immunity in mice with intracranial G422 glioma xenografts. Infiltration of immune cells and the release of inflammatory 203 factors, such as TNF-α and IFN-γ, were increased following PDT when compared to 204 untreated animals [33].

These are all positive results for 5-ALA and PDT in glioma patients or glioma cell cultures. Due to 5-ALA dependent PDT effect that we have observed in three glioblastoma multiforme cell lines (unpublished data) and our control glioma cell line U373, we may speculate that the increased PPIX fluorescence in AtT-20 after 24hrs of incubation in contrast to GH3 leads to a reduction of the proliferation rate and hence to a decline in the Wst-1 signal. Additional irradiation further decreases the signal, but without any statistical significance (Fig 4B). Like most photosensitizers PPIX is fluorescent and this fluorescence progressively decreases during PDT, which is called photobleaching effect [34,35]. Robinson and colleagues could even estimate in normal skin of the SKH HR1 hairless mouse the effect of light dose and fluence rate on the dynamics and magnitude of photobleaching and on the corresponding PDT-induced damage. The rate of PPIX photobleaching is not a simple function of fluence rate, but is dependent on the initial concentration of sensitizer. Furthermore, they could show that PPIX and its photoproducts are not photobleached in the absence of oxygen [36]. Taken together our experiments showed no significant effect of 5-ALA based PDT in the two pituitary adenoma cell lines AtT-20 and GH3 up to concentrations of 200 μg/ml 5-ALA and an incubation period of 24hrs, although we could demonstrate the 5-ALA uptake into the cells. So, the question remains if the applicated fluence rate experienced in further glioma settings is not enough for pituitary adenoma cells in combination with their intracellular photosensitizer concentration, especially without absence of oxygen and possible photobleaching. Due to the difference in PPIX fluorescence, we will have to find out whether the GH3 cells do have an efflux mechanism. The immortalized cell line GH3 does not represent the fully phenotype of pituitary adenoma tissue, but we failed to grow enough primary cell cultures. Thus, further investigations on 5-ALA and its effect on different pituitary adenomas are required.

### Conclusion

Our study demonstrates the 5 ALA induced PPIX fluorescence in two pituitary adenoma cell lines after incubation in-vitro. This might offer surgical possibilities for this method in skull base surgery, helping the surgeon to identify the tumor, enabling a more complete resection and probably improving the clinical outcome. The role of PDT for unresectable pituitary adenomas deserves further investigations.

## Supporting Information

S1 Table U373 cells after 5-ALA incubation and PDT. Cell vitality (%) by Wst-1 testing of U373 glioma cells with incubation of 25 μg/ml 5-ALA and after PDT (n = 12). Control groups were “without 5-ALA and without laser”, “with 5-ALA and without laser” and “without 5-ALA and with laser”. Included are mean values and standard deviation (SD) for each group.
(DOCX)
