# Supplementary material for: 5-ALA Fluorescence in Native Pituitary Adenoma Cell Lines: Resection Control and Basis for Photodynamic Therapy (PDT)?
Source: PLoS One. 2016 Sep 1;11(9):e0161364. doi: 10.1371/journal.pone.0161364 (PMC5008746; doi:10.1371/journal.pone.0161364)
Supplement: S1 Table — Cell vitality (%) by Wst-1 testing of U373 glioma cells with incubation of 25 μg/ml 5-ALA and after PDT (n = 12). Control groups were “without 5-ALA and without laser”, “with 5-ALA and without laser” and “without 5-ALA and with laser”. Included are mean values and standard deviation (SD) for each group. (DOCX) [file pone.0161364.s001.docx]

|  | -ALA25/-Laser | +ALA25/-Laser | -ALA25/+Laser | +ALA25/+Laser |
| --- | --- | --- | --- | --- |
| Cell Vitality (%) | 96,251 | 89,868 | 122,391 | 66,869 |
|  | 93,921 | 90,578 | 115,603 | 68,896 |
|  | 102,330 | 92,908 | 119,453 | 57,345 |
|  | 107,294 | 95,441 | 126,444 | 45,491 |
|  | 101,660 | 98,340 | 102,055 | 55,336 |
|  | 99,842 | 102,767 | 102,846 | 58,103 |
|  | 100,632 | 110,909 | 95,889 | 58,893 |
|  | 97,945 | 103,004 | 100,711 | 54,387 |
|  | 107,040 | 121,957 | 128,878 | 65,632 |
|  | 94,272 | 108,831 | 120,883 | 69,332 |
|  | 97,494 | 118,974 | 109,427 | 57,876 |
|  | 101,313 | 109,905 | 106,324 | 55,251 |
| Mean Value(%) | 100,000 | 103,624 | 112,575 | 59,451 |
| Standard Deviation SD | 4,158 | 10,254 | 10,620 | 6,734 |

S1 Table Supporting File: Cell vitality (%) by Wst-1 testing of U373 glioma cells with incubation of 25 µg/ml 5-ALA and after PDT (n=12). Control groups were “without 5-ALA and without laser”, “with 5-ALA and without laser” and “without 5-ALA and with laser. Included are mean values and standard deviation (SD) for each group.
